# A systematic mini-review of epigenetic mechanisms associated with electroconvulsive therapy in humans

**DOI:** 10.3389/fnhum.2023.1143332

**Published:** 2023-03-09

**Authors:** Sayra Catalina Coral Castro, Carla Bicca, Bruno Bicca, Stéfany Araujo, Thiago Wendt Viola

**Affiliations:** ^1^Developmental Cognitive Neuroscience Lab, School of Medicine, Pontifical Catholic University of Rio Grande do Sul (PUCRS), Porto Alegre, Brazil; ^2^Center for Medical Sciences, Federal University of Pernambuco, Recife, Brazil

**Keywords:** depression, epigenetics, biomarkers, electroconvulsive therapy (ECT), major depression (MD)

## Abstract

**Introduction:**

Electroconvulsive therapy (ECT) is one of the most effective strategies for treating resistant major depression. Although the mechanism of action is not fully understood and studies are limited, epigenetics is a promising area for the development of biomarkers associated with ECT treatment response.

**Aim:**

We reviewed studies available in the literature that explored the epigenetics of ECT in peripheral samples from patients with major depressive disorder (MDD).

**Methods:**

A systematic review was performed following The PRISMA guidelines. The search was performed in seven electronic databases: Scopus, Web of Science, Medline, PsycINFO, Embase, Cochrane, and Cinahl.

**Results:**

Nine studies were included. Seven assessed DNA methylation and three investigated microRNAs (miR). Overall, most studies were exploratory, with small sample sizes, and we found high heterogeneity between the study’s design, ECT protocols, molecular biology methods, and epigenetic findings. Investigated candidates with some evidence of association with ECT treatment response were *BDNF*, *S100A10*, *RNF213M*, *TNKS*, *FKBP5*, miR-126, miR-106a, and miR-24.

**Conclusion:**

The present findings seem to support previous preclinical research, suggesting that epigenetic mechanisms play an important role in the molecular mechanism underlying ECT effects.

## 1. Introduction

Electroconvulsive therapy (ECT) is one of the most effective options for the treatment of pharmacoresistant major depressive disorder (MDD), bipolar disorder that has not responded to other treatments, suicidal behavior, severe agitation, and to manage catatonia and clozapine-resistant schizophrenia (Tor et al., 2021). MDD is one of the most prevalent mental disorders across the world, potentially life-threatening when left untreated. Currently, MDD affects more than 322 million individuals, imposing a considerable economic cost on society (Gutiérrez-Rojas et al., 2020). In addition to the mortality associated with suicidality, the chronic nature of depression contributes substantially to the development of cardiovascular and metabolic illness, increasing the global burden of disease and disability (Sousa et al., 2022).

Deciphering the pathophysiology of depression is a complex phenomenon. Depressive syndromes are not homogeneous, are multidimensional and have multiple etiologies. Treatment with antidepressant drugs is ineffective for 30–50% of these patients and, when effective, it has a delayed onset and considerable side effects (Gaynes et al., 2018). The efficacy of ECT is on average three times greater than pharmacotherapy, offering up to 60% remission of symptoms among patients with MDD, and the therapeutic response is usually faster than existing psychotropic medications (Rhee et al., 2022) However, its therapeutic mechanism of action has not yet been fully elucidated.

Currently, the main findings referring to the mechanism and neurobiological aspects are related to structural, functional and compositional changes in the brain. Based on neuroimaging data, the brain networks involved with ECT include the default-mode network, saliency network, dorsal-attention network, and central-executive network (Wang et al., 2018). These brain networks appear to be valuable biomarkers for assessing the underlying neurobiology of the therapeutic effects of ECT. In addition, decades of research in animal models suggest that ECT exerts its effects through biochemical and molecular changes, including changes in the expression of genes that encode for various transcription factors, neurotrophins and structural proteins, as well as the alteration in cerebral blood flow, blood-brain barrier, neurotransmitters and neuroinflammatory signaling (Réus et al., 2019). This suggests that peripheral molecular markers also possess the potential to elucidate the mechanisms underlying ECT.

Epigenetic modifications such as DNA methylation, histone alterations, and the functional role of non-coding RNAs are potentially reversible molecular processes that can induce lasting changes in gene expression (Bredy et al., 2010). This is particularly relevant considering ECT effects, once epigenetic mechanisms might be rapid and long-lasting, providing fine-tune regulation of experience-induced gene expression (Dudley et al., 2011; Baker-Andresen et al., 2013). However, the current evidence explaining the possible role of ECT in modifying epigenetic mechanisms with therapeutic effects is mostly restricted to preclinical studies (Jiang et al., 2017). Furthermore, little is known about the implications of ECT on the epigenetic machinery in patients with MDD. Identifying reliable molecular biomarkers that could predict response to ECT would significantly reduce costs and identify patients eligible for first-line ECT treatment. Hence, we performed a systematic mini-review of the literature regarding ECT and epigenetics, focusing entirely on human studies.

## 2. Materials and methods

This review was performed following The PRISMA guidelines (Moher et al., 2009). The research question was: *Is electroconvulsive therapy associated with changes in epigenetic markers?* For this, on 17 December 2020, we used the following descriptors (MiRNAs OR microRNAs OR epigenetic OR epigenomic OR epigenomics OR DNA methylation OR 5 mC OR DNA hydroxymethylation OR 5 hmC OR histone acetylation OR histone deacetylation OR histone methylation OR long non-coding RNA OR lincRNA OR epigenetic process) AND (electroconvulsive OR ECT OR electroconvulsive therapy) to search in seven electronic databases: Scopus, Web of Science, Medline, PsycINFO, Embase, Cochrane, and Cinahl.

After duplicate removal, three independent researchers screened search records by title and abstract, using the Rayyan QCRI software (Ouzzani et al., 2016). The inclusion criteria were: studies with samples of men/women between 18 and 65 years old, with MDD and psychiatric indication for treatment with ECT; studies who had any epigenetic investigation associated with ECT. Observational studies randomized controlled trials (RCT), cross-sectional, and case-control studies were considered; while qualitative studies, animal models, summaries, book chapters, literature reviews, or studies that did not present any epigenetic investigation were excluded. Occasional divergence in selection was resolved by consensus with the help of our mentor.

Full-text studies were evaluated in detail in terms of the objective, hypothesis, and study design. From a methodological perspective, samples were characterized (sample size, psychiatric diagnosis, etc.) and the clinical scales used to diagnose patients were also evaluated. Mechanical and functional scrutiny of the ECT protocol was carried out, seeking to specify the duration (number of sessions), frequency (number of weekly or monthly sessions), equipment used (type of device), applied anesthesia and electrical stimulation, unilateral or bilateral, lower/higher dosages. Regarding epigenetic outcomes, we focused mainly on molecular biology methods (analyses and extraction of DNA or RNA) and on which epigenetic markers were evaluated (DNA modifications, non-coding RNA, etc.).

## 3. Results

### 3.1. Characteristics of studies

**FIGURE 1:**
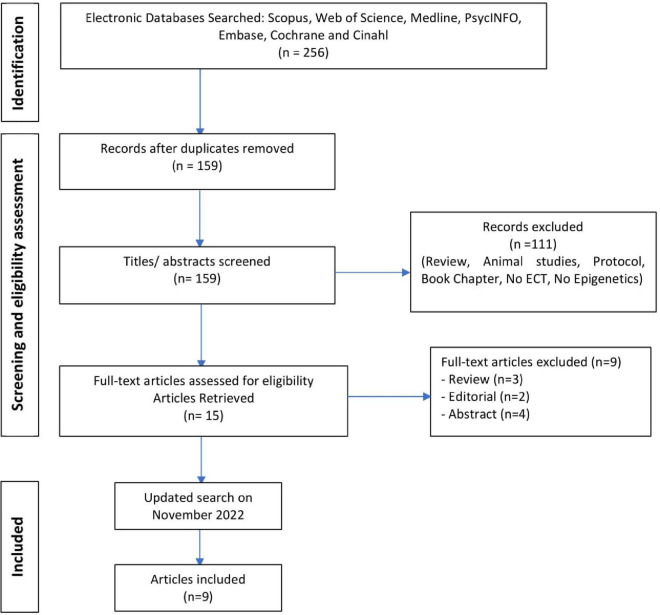
Preferred Reporting Items for Systematic Reviews (PRISMA) Flow Diagram. The detailed process for selecting articles evaluating the association between epigenetic processes and electroconvulsive therapy (ECT) in humans.

The nine reviewed studies combined for a sample of 358 participants with MDD (mean clinical sample size = 39). Figure 1 displays the flowchart of the screening and eligibility process. Characteristics of studies are presented in Table 1. Studies were published between 2014 and 2022.

**TABLE 1:** Study design and epigenetic findings.

References	Sample	Psychopharmacotherapy	Measures	ECT protocol	Molecular biology methods	Findings
Kleimann et al., 2015	Eleven patients with TRD receiving ECT treatment: 4 patients were in remission and 6 patients had responded to ECT	Antidepressant (no specification) Atypical antipsychotic	SCID-1 MADRS BDI-II	-Duration: 3–5 weeks -Frequency: three times a week -Equipment: Thymatron IV brief-pulse device (Somatics; Lake Bluff, IL, USA) -Anesthesia: n/a	Sample type: genomic DNA extracted from whole blood Targeted bisulfite sequencing of DNA methylation of *BDNF* promoter I, IV, VI	ECT responders had a significantly lower promoter mean methylation rate compared to non-responders Analysis of the individual promoter methylation rates revealed that this effect is mainly driven by methylation of *BDNF* promoter I
Gururajan et al., 2016	Forty patients with TRD: 24 treated with ECT; 16 treated with Ketamine. 20 healthy controls	SSRI NRI SNRI Bupropion TCA SARI NaSSA	HDRS (HAM-D)	-Duration: n/a -Frequency: bi-weekly -Equipment: brief-pulse bitemporal -Anesthesia: n/a	Sample type: total RNA extracted from whole blood microRNA transcriptome through microarray Validation by quantitative PCR	The baseline expression of let-7b and let-7c were significantly lower in all patients compared with healthy controls Baseline expression of let-7b and let-7c did not predict treatment response, and their expression did not significantly changed after ECT
Kolshus et al., 2017	Discovery phase: deep sequencing study: 16 patients (before and after ECT) with moderate-to-severe MDD Validation phase: 37 (14 responders and 23 non-responders) patients MDD and 34 healthy controls	SSRI SNRI Tetracyclic antidepressant Bupropion Agomelatine Lithium Antipsychotic Benzodiazepine Anticonvulsivant Hypnotic	SCID-1 HDRS (HAM-D) BPRS	-Duration: n/a -Frequency: twice weekly -Equipment: hand-held electrodes on the Spectrum 5000 M device (Mecta Corp., Oregon, USA) with a maximum output of 1200 millicoulombs (mC) -Anesthesia: Methohexitone (0.75–1.0 mg/kg), a short-acting barbiturate and suxamethonium (0.5–1.0 mg/kg) as a muscle relaxant	Sample type: total RNA extracted from whole blood Small RNAs NGS (microRNA transcriptome) Validation-phase pre-/post-ECT: qRT-PCR Validation-phase case-control: qRT-PCR	Sequencing study found miR-130a-3p/5p, miR-126-3p/5p, miR-106a-5p, and miR-942-3p as potential targets associated with ECT In validation, there were no statistically significant differences in the levels of selected miRNAs following ECT Among a subgroup of patients with psychotic depression, decreased levels of miR-126-3p and miR-106a-5p were found after ECT
Neyazi et al., 2018	Discovery phase: 11 patients with MDD receiving ECT Validation phase: 67 patients with MDD receiving ECT	SSRI	MADRS HDRS (HAM-D)	-Duration: 6 weeks -Frequency: once or twice weekly -Equipment: n/a -Anesthesia: n/a	Sample type: genomic DNA extracted from whole blood Targeted bisulfite sequencing of *S100A10* (p11) promoter DNA methylation	Higher *S100A10* promoter DNA methylation was found in responders to ECT *S100A10* hypermethylation predicted ECT response with a probability of 89%
Moschny et al., 2020a	Seventeen TRD patients: 10 responders and 7 non-responders	Antidepressant (no specification) Benzodiazepine Antipsychotic Lithium	MADRS ICD-10 BDI-II MMSE	-Duration: 4 weeks -Frequency: once a week -Equipment: right unilateral electrical stimulation was performed using an ultra-brief impulse device (Thymatron™ System IV, Somatics, LLC) -Anesthesia: methohexital [mean = 128.2 (±53.3) mg, minimum = 90 mg, maximum = 250 mg] and remifentanil [89.7 (±49.8) mg, 30 mg, 200 mg] and received succinylcholine for muscle relaxation [114.1(±45.0) mg, 60 mg, 200 mg]	Sample type: genomic DNA extracted from PBMC Methyl Capture EPIC NGS: Genome-wide DNA methylation	DNAm analysis of 1476812 single CpG sites revealed five novel (protein-coding) candidate genes to be implicated in ECT response (*RNF175, RNF213, TBC1D14, TMC5*, and *WSCD1*). The top hit was *RNF213* with four CpG sites ECT respondent group also showed differences within regions of long non-coding RNA transcripts (AC018685.2, AC098617.1, and CLCN3P1)
Moschny et al., 2020b	Fifty-nine patients with MDD were assessed at the beginning of ECT treatment. Multiple time points were evaluated in 28 of them	Antidepressant (no specification) Benzodiazepine Antipsychotic	HDRS (HAM-D) MADRS ICD-10 BDI-II MMSE	-Duration: 4 weeks -Frequency: three times a week -Equipment: Ultra-brief pulse devices [Mecta 5000Q and Thymatron™ System IV (Somatic LLC)] -Anesthesia: propofol, or etomidate, or methohexital and remifentanil. Succinylcholine served for muscle relaxation	Sample type: genomic DNA extracted from whole blood and from PBMC Targeted bisulfite sequencing of *PLAT* (t-PA) and *SERPINE1* (PAI-1) promoter DNA methylation	No detectable DNA methylation differences between ECT remitters and non-remitters. However, DNA methylation differed greatly between the different immune cells
Israel-Elgali et al., 2021	Forty-seven patients with TRD: 17 were treated with ECT; 14 were treated with Ketamine; 16 were treated as usual. 23 healthy controls	SSRI SNRI Antipsychotic	HDRS (HAM-D) HAMA QIDS-SR	-Duration: 3 weeks -Frequency: twice weekly -Equipment: ECT was conducted with a Thymatron IV device -Anesthesia: propofol (1.20–2.69 mg/kg) for anesthesia and succinylcholine (0.45–1.09 mg/kg) as a muscle relaxant	Sample type: miRNA extracted from PBMC NanoString nCounter for microRNA transcriptome	Adjustment of *p*-values for genome-wide profiling did not yield any statistically significant changes in miRNA levels By analyzing data with less statistical stringency, an upregulation of miR-24-3p levels was found associated with ECT
Sirignano et al., 2021	Thirty-four TRD patients receiving ECT treatment: 25 responded to treatment; 9 did not respond to treatment	Antidepressant (no specification)	HDRS (HAM-D) ICD-10	-Duration: 5–25 sessions -Frequency: 2–3 sessions per week -Equipment: hand-held electrodes -Anesthesia: s-ketamine (∼1.0 mg/kg) 25, 26 and succinylcholine for muscle relaxation (∼1.0 mg/kg)	Sample type: genomic DNA extracted from whole blood The Infinium Methylation EPIC Array: Genome-wide DNA methylation	*TNKS and FKBP5* were the only differentially methylated genes with a statistical threshold of a *p*-value × 10^–7^ associated with ECT response
Schurgers et al., 2022	Nineteen TRD Patients with unipolar or bipolar depression	SNRI TCA Trazodone Bupropion MAOI Agomelatine Lithium Antipsychotic Benzodiazepine Anticonvulsivant	HDRS (HAM-D) BDI-II DSM-IV	-Duration: 3–11 sessions -Frequency: 2 sessions per week -Equipment: Thymatron System IV device (Somatics, LLC, Lake Bluff, IL, USA), bitemporal electrode position -Anesthesia: etomidate (0.1–0.2 mg/kg) and for muscle relaxation succinylcholine (0.5–1.0 mg/kg)	Sample type: genomic DNA extracted from whole blood The Infinium Methylation EPIC Array (Targeted genes found in gene expression experiment): DNA methylation of *BDNF, ERK1* and *NR3C1*	Blood mRNA levels of *BDNF*, *ERK1*, and *NR3C1* increased during ECT treatment, and significant correlations between mRNA levels and DNA methylation levels were found for all the three investigated genes

n/a, information was not available/not reported; ECT, electroconvulsive therapy; MDD, major depressive disorder; TRD, treatment-resistant depression; PBMC, peripheral blood mononuclear cells; mRNA, microRNA; SCID-1, structured clinical interview for the DSM-IV axis 1 disorders; HDRS, hamilton depression rating scale; BPRS, brief psychiatric rating scale; CMS, chronic mild stress model; MADS, montgomery asberg depression scale; HAMD, hamilton depression rating scale; ICD-10, international statistical classification of diseases and related health problems 10th revision; BDI-II, beck depression inventory; MADRS, mont-gomery-åsberg depression rating scale; MMSE, mini-mental state examination; QIDS-SR, quick inventory of depressive symptomatology-selfReport; TAU, treatment as usual; KET, ketamine; BDNF, brain derived neurotrophic factor; PBMC, peripheral blood mononuclear cells; SSRI, selective serotonin reuptake inhibitor; NRI, norepinephrine reuptake inhibitor; SNRI, serotonin and noradrenaline reuptake inhibitor; SARI, serotonin antagonist and reuptake inhibitor; NaSSA, noradrenergic and specific serotonergic antidepressant; TCA, tricyclic antidepressant; MAOI, monoamine oxidase inhibitor; NGS, next-generation sequencing.

All were performed with patients with MDD (Kolshus et al., 2017; Neyazi et al., 2018; Moschny et al., 2020a), while six studies referred to cases of treatment-resistant depression (TRD) (Kleimann et al., 2015; Gururajan et al., 2016; Moschny et al., 2020b; Israel-Elgali et al., 2021; Sirignano et al., 2021; Schurgers et al., 2022). They were all observational studies of a cohort of patients enrolled in a clinical trial with ECT, and with the evaluation of longitudinal biomarkers. Two studies also evaluated epigenetic changes induced by ketamine in addition to ECT (Gururajan et al., 2016; Sirignano et al., 2021). Some studies compared patients who improved from depression after ECT with those who did not improve (Kleimann et al., 2015; Kolshus et al., 2017; Moschny et al., 2020b; Sirignano et al., 2021), while some studies compared longitudinal changes before and after ECT treatment (Gururajan et al., 2016; Neyazi et al., 2018; Moschny et al., 2020a; Israel-Elgali et al., 2021; Schurgers et al., 2022).

Heterogeneity was found regarding ECT protocols. Treatment lasted from 2 to 6 weeks. The frequency was one to three times a week. Regarding the ECT method, brief-pulse was the most used method. The devices used were Spectrum Mecta^®^ 5000 (Kolshus et al., 2017; Moschny et al., 2020a), and Thymatron^®^ System IV (Kleimann et al., 2015; Moschny et al., 2020b; Israel-Elgali et al., 2021; Schurgers et al., 2022). Most studies reported the usage of short-acting anesthetics. The application of the electrical stimulus was unilateral (Moschny et al., 2020b) or bitemporal (Gururajan et al., 2016; Schurgers et al., 2022). Psychiatric pharmacotherapy is presented in detail in Table 1.

### 3.2. Effects of ECT on microRNAs

Epigenetic findings of studies are summarized in Table 1. MicroRNAs (miRNAs) are a class of non-coding RNAs, averaging 22 nucleotides in length, that play important roles in regulating gene expression (Jonas and Izaurralde, 2015). These small molecules accomplish essential post-transcriptional regulatory steps of gene expression through either the degradation of an mRNA transcript or the inhibition of translation (He and Hannon, 2004). Both in the peripheral and central nervous systems, miRNAs are considered potential biomarkers in the diagnosis of MDD and in monitoring therapeutic responses (Šalamon Arèan et al., 2022). They regulate key processes in the central nervous system including neuronal development, neurogenesis, and synaptic plasticity, while in the periphery they are implicated with immune and endocrine regulation, for instance.

Three studies assessed miRNAs in peripheral blood or peripheral blood mononuclear cells (PBMCs). Gururajan et al. (2016) investigated by microarray the expression of several blood miRNAs at 2 different time points, before and after ECT. In addition, they assessed miRNAs in healthy controls. Pre-ECT expression of let-7b and let-7c was lower in TRD patients compared to controls. However, pre- and post-treatment levels were similar, and baseline expression of these miRNAs could not predict ECT treatment response. These results suggest that let-7b and let-7c miRNAs may be useful as diagnostic biomarkers of TRD, but not of ECT. Kolshus et al. (2017) performed a transcriptomic analysis through RNA-sequencing of miRNAs in the blood of patients after ECT, finding miR-130a-3p/5p, miR-126-3p/5p, miR-106a-5p, and miR-942-3p as potential targets associated with ECT response, but these findings were not confirmed in the validation study in a larger sample. Interestingly, in the validation study, they found that miR-126-3p and miR-106a-5p were significantly elevated in patients with psychotic depression in baseline when compared to healthy controls. However, after ECT the levels of these miRNAs normalized. Finally, Israel-Elgali et al. (2021) analyzed RNA samples extracted from PBMCs from patients with TRD submitted to ECT. By using NanoString technology and assessing hundreds of miRNAs, they found no statistically significant changes in miRNAs as a result of ECT.

### 3.3. Effects of ECT on DNA methylation

DNA methylation is a major epigenetic mechanism that regulates gene expression by recruiting proteins involved in gene repression or by inhibiting the binding of transcription factors to DNA. This epigenetic mechanism involves the transfer of a methyl group onto the C5 position of the cytosine to form 5-methylcytosine, frequently occurring on cytosines followed by a guanine nucleotide, known as CpG sites (Moore et al., 2013). The methylation of nucleobases can change the functionality of a segment of DNA without altering the sequence. Studies have shown altered DNA methylation patterns associated with MDD and antidepressants (Webb et al., 2020; Šalamon Arèan et al., 2022).

Six studies evaluated the relationship between DNA methylation and the effects of ECT (Kleimann et al., 2015, Neyazi et al., 2018; Moschny et al., 2020a,b; Sirignano et al., 2021; Schurgers et al., 2022). DNA samples were extracted from peripheral blood in all studies. Kleimann et al. (2015) investigated the methylation levels of the *BDNF* gene promoter in 11 TRD patients before and during ECT sessions. Responders had lower methylation rates, especially in the exon I promoter of *BDNF*. In addition, Schurgers et al. (2022) found that the gene expression of *BDNF*, *ERK1*, and *NR3C1*, in blood samples of TRD patients, significantly increased over time during ECT treatment. For these 3 genes changes, expression levels were highly correlated with changes in DNA methylation levels for several CpG sites, including negative correlations between mRNA levels of *BDNF* and methylation (i.e., hypomethylation), particularly in 3 CpGs (cg15462887, cg27351358, cg23426002). This suggests some overlap between the findings of Schurgers and Kleimann’s studies, at least regarding *BDNF* demethylation throughout ECT treatment. Neyazi et al. (2018) assessed DNA methylation of the p11 promoter, also known as the *S100A10* gene. The protein coded is multifunctional, and previous evidence suggests a link between depressive states, and response to antidepressant treatment, with peripheral p11 levels (Chen et al., 2022; Gałecka et al., 2022). Interestingly, they found higher *S100A10* promoter DNA methylation in responders to ECT. Moschny et al. (2020b), on the other hand, investigated DNA methylation of the promoter regions of the genes *PLAT* and *SERPINE1*, finding no significant differences between baseline and post-ECT DNA methylation levels after controlling for potential confounders.

Different from the aforementioned studies that used candidate gene approaches, Moschny et al. (2020a) identified 5 genes (*RNF175, RNF213, TBC1D14, TMC5*, and *WSCD1*) and 3 non-coding RNAs (*AC018685.2, AC098617.1*, and *CLCN3P1*), differentially methylated, using a genome-wide approach in samples of TRD patients undergoing ECT. The highest effect sizes were found in four CpG sites located in the *RNF213* gene in the group that responded to ECT. Finally, Sirignano et al. (2021), examined treatment-associated changes in DNA methylation levels in 34 patients across the epigenome. Among the 20 genes with a significant effect, *TNKS* and *FKBP5* were the only differentially methylated genes with a *p*-value below 10^–7^, highlighting their potential for future investigations.

## 4. Discussion

ECT was introduced more than 80 years ago and was one of the early biological treatments for mental disorders. Epigenetics is one of the areas that can help to better understand the molecular processes associated with ECT action. Genes are regulated through the activity of epigenetic mechanisms, serving to turn up or turn down levels of gene expression in response to rapidly changing stimuli, such as ECT. In this review, we included nine studies that investigated either miRNAs or DNA methylation patterns related to ECT in peripheral samples of MDD patients. Overall, most studies were exploratory, with small sample sizes, and we found high heterogeneity between the study’s design, ECT protocols, molecular biology methods, and epigenetic findings.

DNA methylation was interrogated in 6 studies. Four of them had a candidate gene approach (Kleimann et al., 2015; Neyazi et al., 2018; Moschny et al., 2020b; Schurgers et al., 2022), focusing on genes that have been frequently associated with the molecular pathophysiology of MDD, such as *BDNF* and *NR3C1*. Although preliminary and without overlap regarding the assessed CpGs on DNA sequence, Kleimann et al. (2015) and Schurgers et al. (2022) presented evidence suggesting that ECT response is associated with a demethylation pattern of *BDNF* (e.g., exon 1 promoter). If considering that demethylation might be associated with higher gene expression, such as partially showed by Schurgers et al. (2022), these findings are promising. Particularly, enhancement of the BDNF signaling through epigenetic regulation and increased gene expression has been suggested to be a molecular mechanism of action of conventional antidepressants (Cubillos et al., 2022). However, it should be noted that the 2 methylome studies included in our review (Moschny et al., 2020a; Sirignano et al., 2021), did not find *BDNF* as a significantly differentially methylated gene regarding ECT. Similarly, a hypermethylation pattern of the *S100A10* promoter in responders to ECT was found at the level of targeted genes by Neyazi et al. (2018), but this was not confirmed by the methylome studies. Hence, when analyzing the findings of the 2 genome-wide DNA methylation studies included in our review, several promising hits were discovered. By applying the Illumina Infinium Methylation EPIC Array, or the Methyl Capture EPIC Next-generation sequencing, these studies analyzed thousands of CpG sites across the genome. Therefore, Moschny et al. (2020a) identified non-coding RNAs, in addition to protein-coding genes, as potential genomic regions with DNA methylation patterns associated with ECT treatment response. In addition, Sirignano et al. (2021) found *FKBP5* as one out of two differentially methylated genes with a stringent statistical significance threshold associated with ECT. *FKBP5* is a well-known gene involved in neuroendocrine signaling associated with MDD, supported by both genetic and epigenetic evidence (Alshaya, 2022). Changes in *FKBP5* gene expression in the blood are also associated with ECT treatment response (Israel-Elgali et al., 2021). Although the findings of these genome-wide studies require further validation, it seems clear that unbiased methods of epigenetic investigation are the most promising strategies for revealing ECT molecular biomarkers.

In this sense, all studies that assessed the relationship between ECT and miRNAs performed transcriptomic analyses, not focusing entirely on candidate miRNAs. Therefore, several miRNAs emerged as potential biomarkers of ECT response, requiring further validation. The most relevant considering statistical significance thresholds and longitudinal changes pre- and post-ECT treatment are miR-126, miR-106a, and miR-24. These 3 miRNAs have already been associated with depressive disorders, by evidence showing altered levels in peripheral blood samples of patients with MDD (Camkurt et al., 2020; Maffioletti et al., 2021; Roumans et al., 2021). It seems that ECT treatment response is associated with a pattern of normalization of their expression in blood samples, which are altered by MDD in the pre-ECT period. The mRNA molecules that these miRNAs target and regulate post-transcriptionally as a function of ECT still need to be investigated.

This review has limitations that should be discussed. First, we only included nine studies, showing that the relationship between ECT and epigenetics is still lacking robust evidence when investigated in humans. Second, most studies were performed with small sample sizes, and their findings need validation in larger samples. This is particularly relevant for genome-wide studies since they usually need high statistical power and larger sample sizes to deal with stringent false discovery rate, as well as with cell-type deconvolution algorithms. Importantly, cell-type-specific data is critical for the proper interpretation of epigenetic findings (Avila Cobos et al., 2020). Third, although DNA methylation and miRNA transcriptional levels were assessed mostly in whole blood samples, ECT is applied in the brain, suggesting that peripheral molecular mechanisms may not overlap with central ones. Fourth, the possible effects of anesthesia and pharmacotherapy are potential confounders, especially in severely depressed patients receiving multiple antidepressant regimens. Few studies have explored the relationship with pharmacotherapy, except for those investigating ketamine versus ECT (Gururajan et al., 2016; Sirignano et al., 2021). Finally, another important group of epigenetic modifications, and for which we found no study, is the covalent post-translational modification of histone proteins. Evidence suggesting that histone modifications are part of the epigenetic landscape associated with ECT effects is still restricted to animal studies (Pusalkar et al., 2016).

Despite these limitations, the present findings seem to support previous preclinical research (Dyrvig et al., 2015), suggesting that epigenetic mechanisms play an important role in the molecular mechanism underlying ECT effects. With changes in the public perception of ECT and more funding for this area of research, these preliminary findings could be deeper explored and validated by future research.

## Author contributions

SC, CB, BB, and SA performed all steps of the systematic review, identification, screening, eligibility, and data extraction. TV supervised all steps, solved any discrepancy, and reviewed the manuscript. SC was the major contributor in writing, while CB, BB, SA, and TV also assisted in writing. All authors contributed to the article and approved the submitted version.
